# Supplementary material for: In-situ adsorption-coupled-oxidation enabled mercury vapor capture over sp-hybridized graphdiyne
Source: Nat Commun. 2025 Mar 11;16:2439. doi: 10.1038/s41467-025-57197-8 (PMC11897331; doi:10.1038/s41467-025-57197-8)
Supplement: Supplementary file 1 — Supplementary Information [file 41467_2025_57197_MOESM1_ESM.pdf]

Supplementary Information

In-situ adsorption-coupled-oxidation enabled mercury vapor capture over *sp*-hybridized graphdiyne

Honghu Li[1,2,ǂ] *Chuanqi Pan*[1,3,ǂ] *Xiyan Peng*[1,2], *Biluan Zhang*[1,3], *Siyi Song*[1,3], *Ze Xu*[1,3], *Xiaofeng Qiu*[1,3], *Yongqi Liu*[1,3], *Jinlong Wang*[1,3,4], *Yanbing Guo*[1,3,4]*

[1]Institute of Environmental and Applied Chemistry, College of Chemistry, Central China Normal University, Wuhan 430079, P. R. China

[2]Research Center for Environment and Health, School of Information Engineering, Zhongnan University of Economics and Law, Wuhan, Hubei 430073, P. R. China

[3]Engineering Research Center of Photoenergy Utilization for Pollution Control and Carbon Reduction, Ministry of Education, College of Chemistry, Central China Normal University, Wuhan 430079, P. R. China

[4]Wuhan Institute of Photochemistry and Technology, Wuhan 430082, P. R. China

ǂH. Li and C. Pan contributed equally to this work.

*E-mail: guoyanbing@mail.ccnu.edu.cn

The Supplementary Information contains Supplementary Figures (23 figures), Supplementary Tables (8 tables), Supplementary Notes and Supplementary References.

Supplementary Figures

Supplementary Fig. 1. The structure of GDY and its theoretical performance. a The molecular structure of GDY; **b** The 2D charge distribution of GDY; **c** GDY adsorbs multiple Hg atoms at the same quality (72 carbon atoms); **d** The atomic model and configuration of 3GDY; **e** The diffusion path of Hg atoms in 3GDY; **f** 3GDY adsorbs multiple mercury atoms (left figure for top view and right figure for side view).

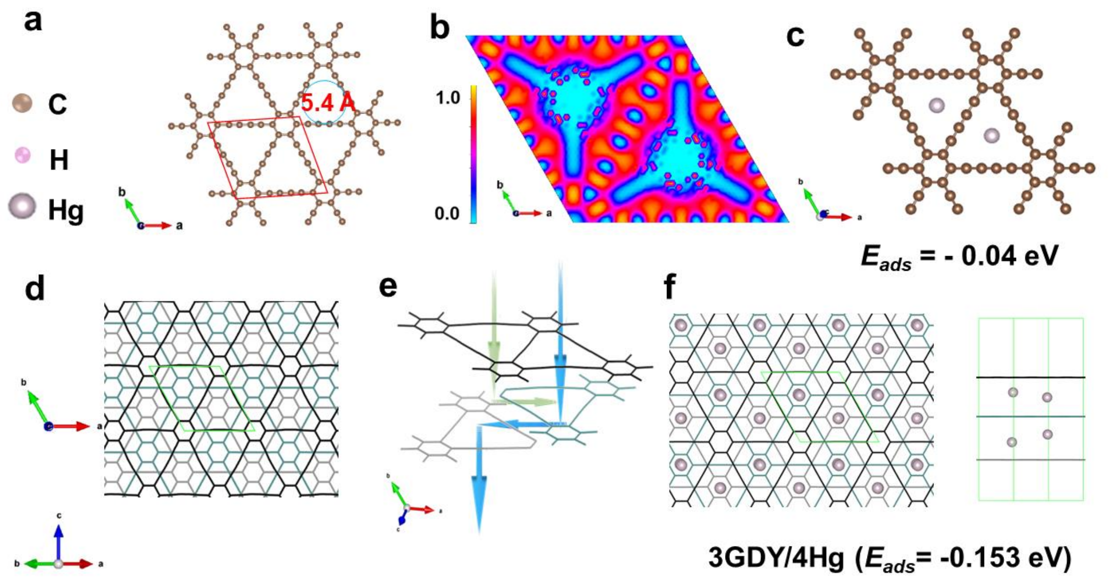

Supplementary Fig. 2. The atomic model and configuration of the different carbon materials. a HsGDY (hydrogen substituted graphdiyne), **b** GDY (graphdiyne), **c** GE (graphene) and **d** SWCNT (single-wall carbon nanotube).

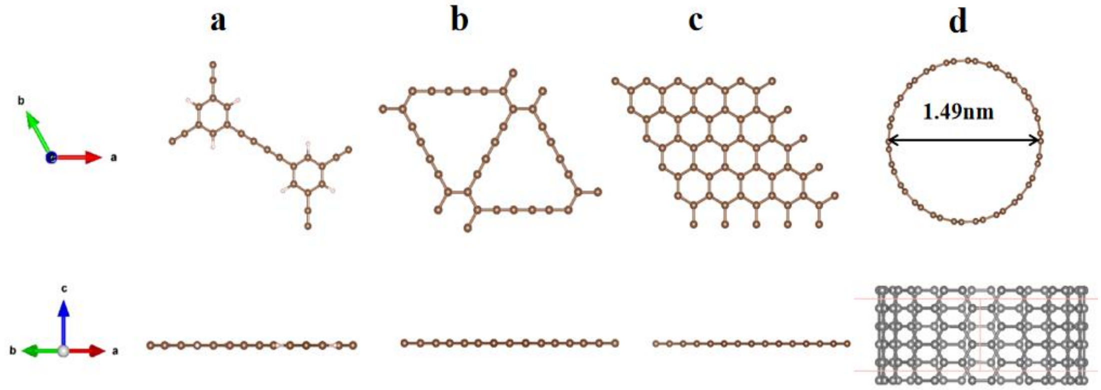

Supplementary Fig. 3. Charge non-uniformity comparison. **a** calculation method for HsGDY, **b** calculation method for GDY and **c** results of comparing the charge non-uniformity of GDY and HsGDY.

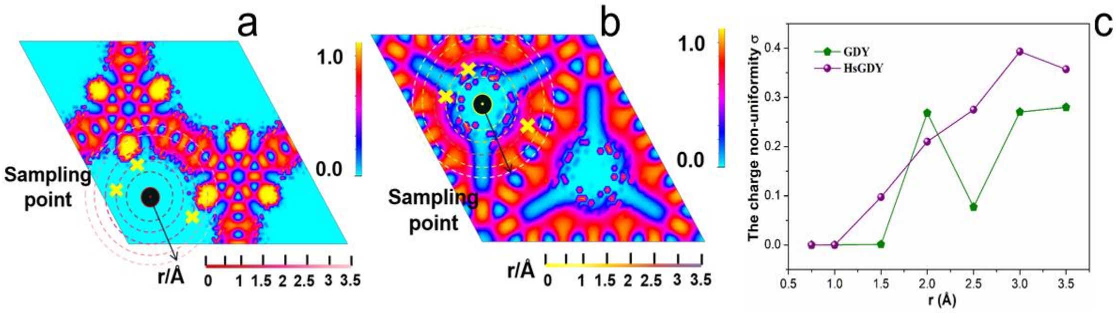

Supplementary Fig. 4. The Morphology and pore characteristic of HsGDY. a SEM image, **b** N_2 adsorption/desorption curves and **c** pore distribution.

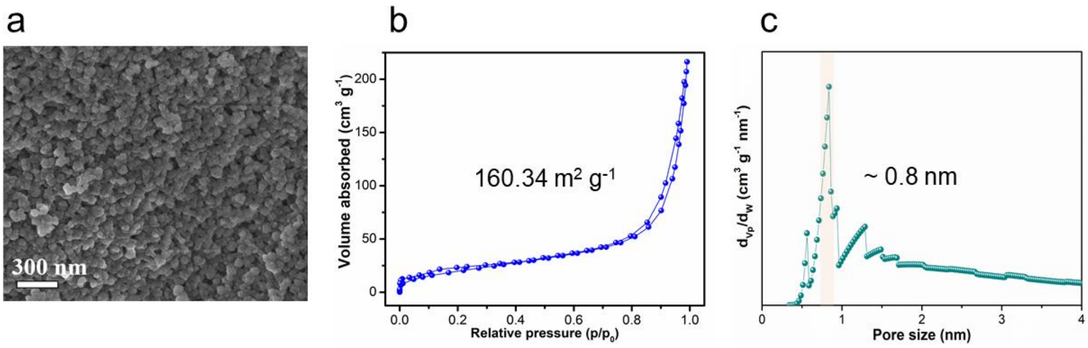

Supplementary Fig. 5. Schematic diagram of the lab-scale fixed-bed reaction system (RightsLink license from Elsevier Ltd.).

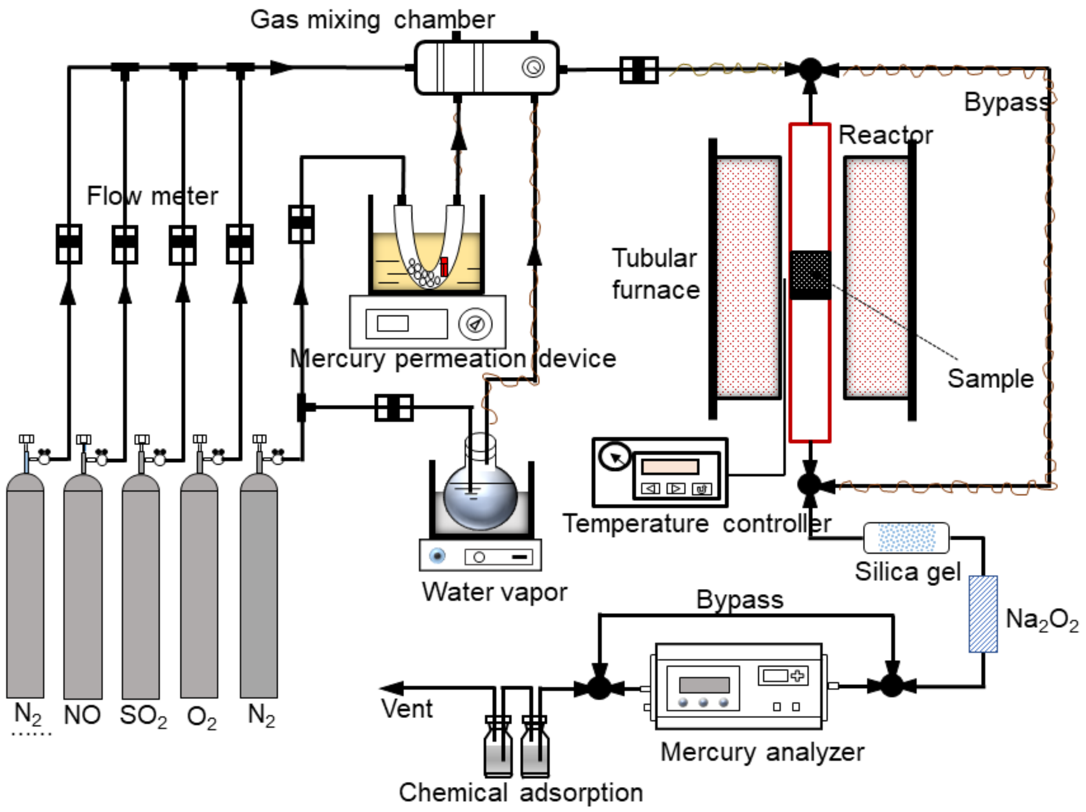

Supplementary Fig. 6. The variation of Hg^0 adsorption capacity with average pore volume and pore width. a with pore volume and **b** with pore width.

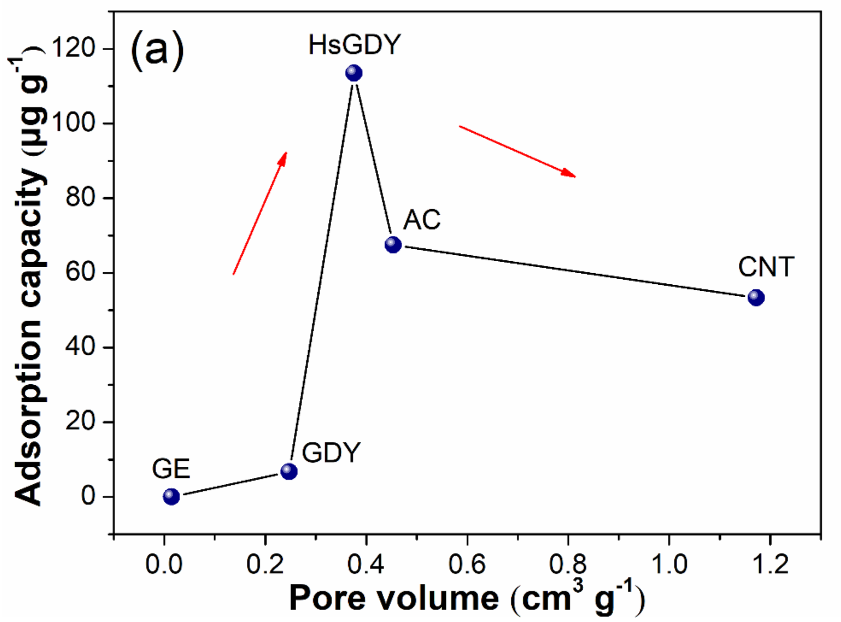

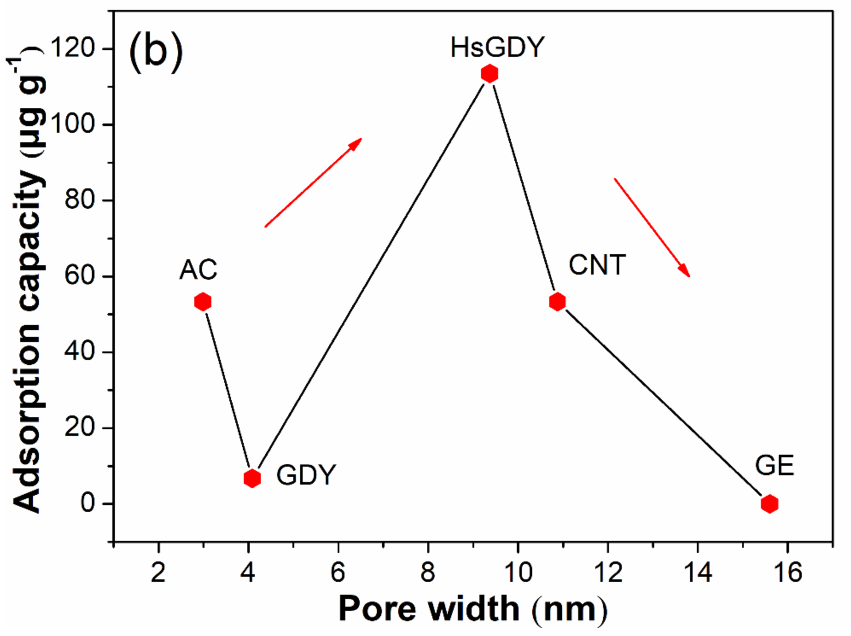

Supplementary Fig. 7. The 50%-breakthrough curve of HsGDY for Hg^0 capture (dash line indicates time point for gas flow passing through sorbent).

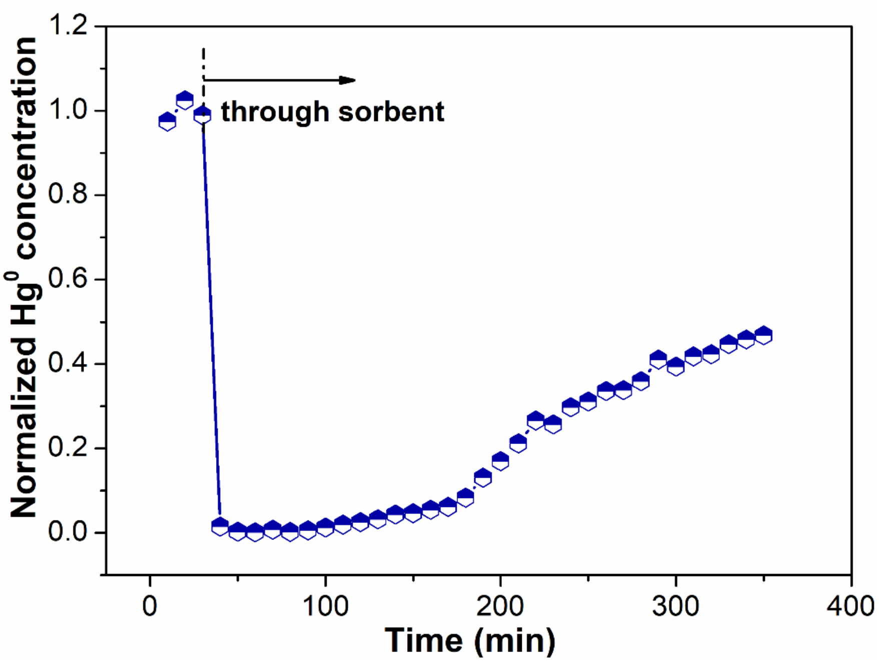

Supplementary Fig. 8. Kinetic analysis. a pseudo-first order model and **b** Weber-Morris model.

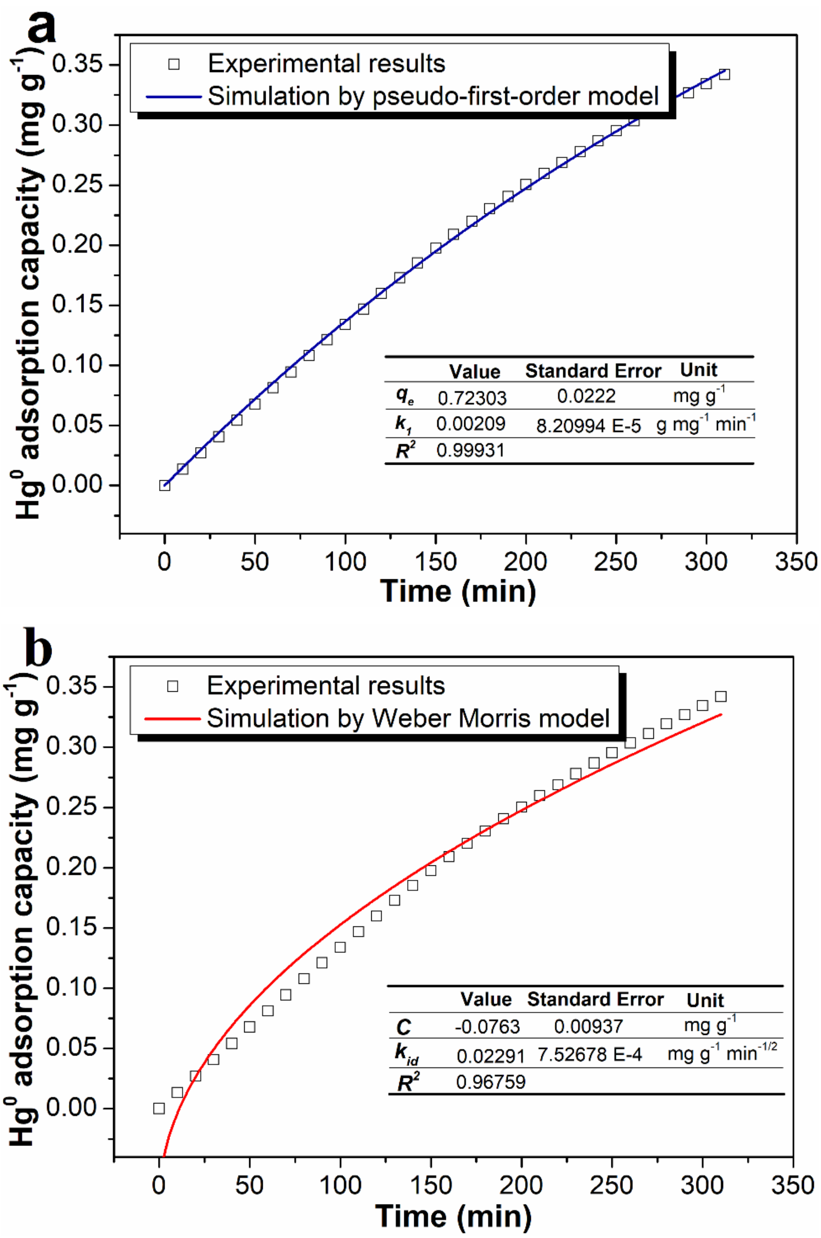

Supplementary Fig. 9. The Hg-TPD results of GDY and HsGDY (heating rate = 2 °C min^{-1}).

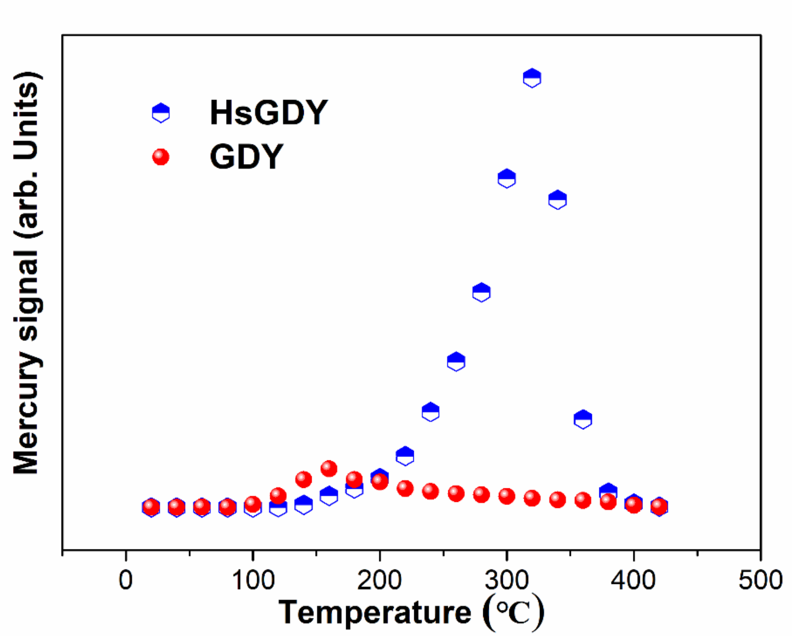

Supplementary Fig. 10. Effects of gas components on Hg^0 adsorption over HsGDY. a effect of NO, **b** effect of H_2O and **c** effect of CO.

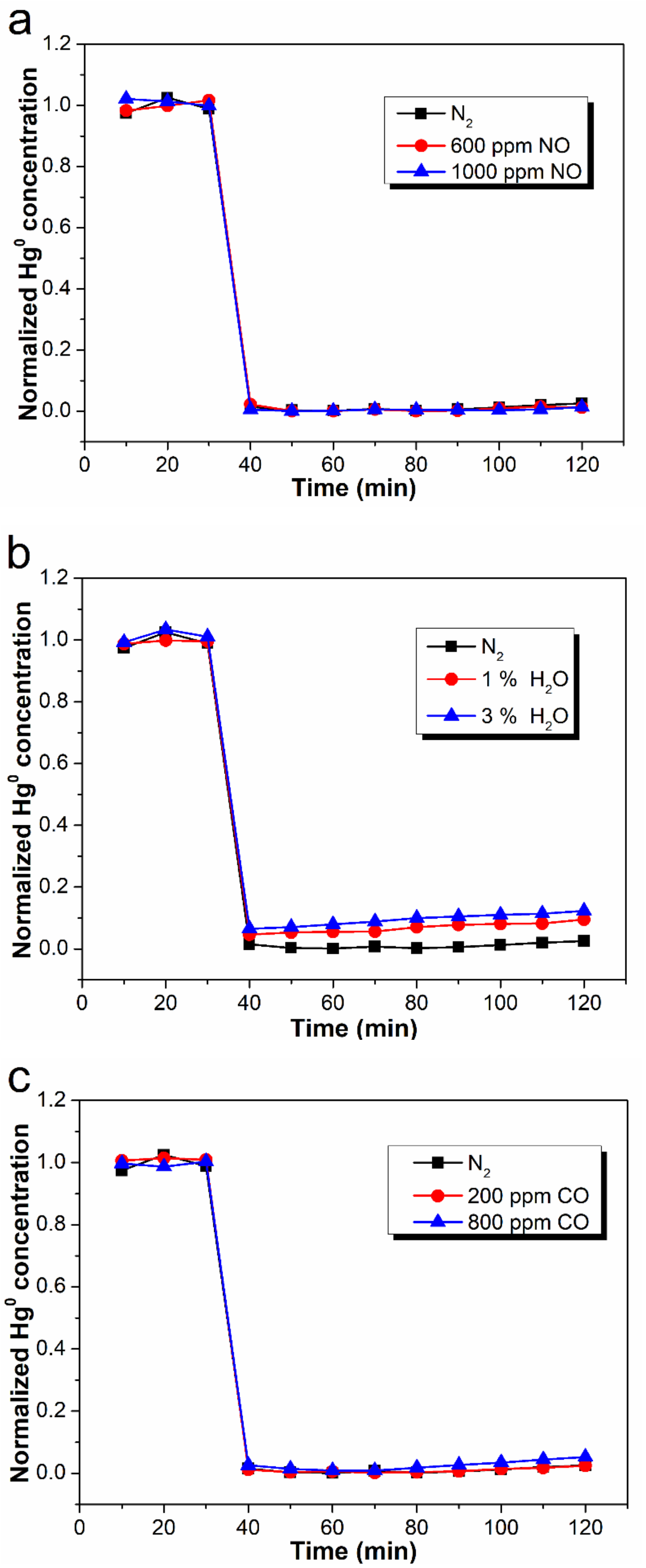

Supplementary Fig. 11. The possible adsorption configurations and adsorption energies of different gas molecules onto HsGDY. a SO_2, **b** NO, **c** H_2O and **d** CO.

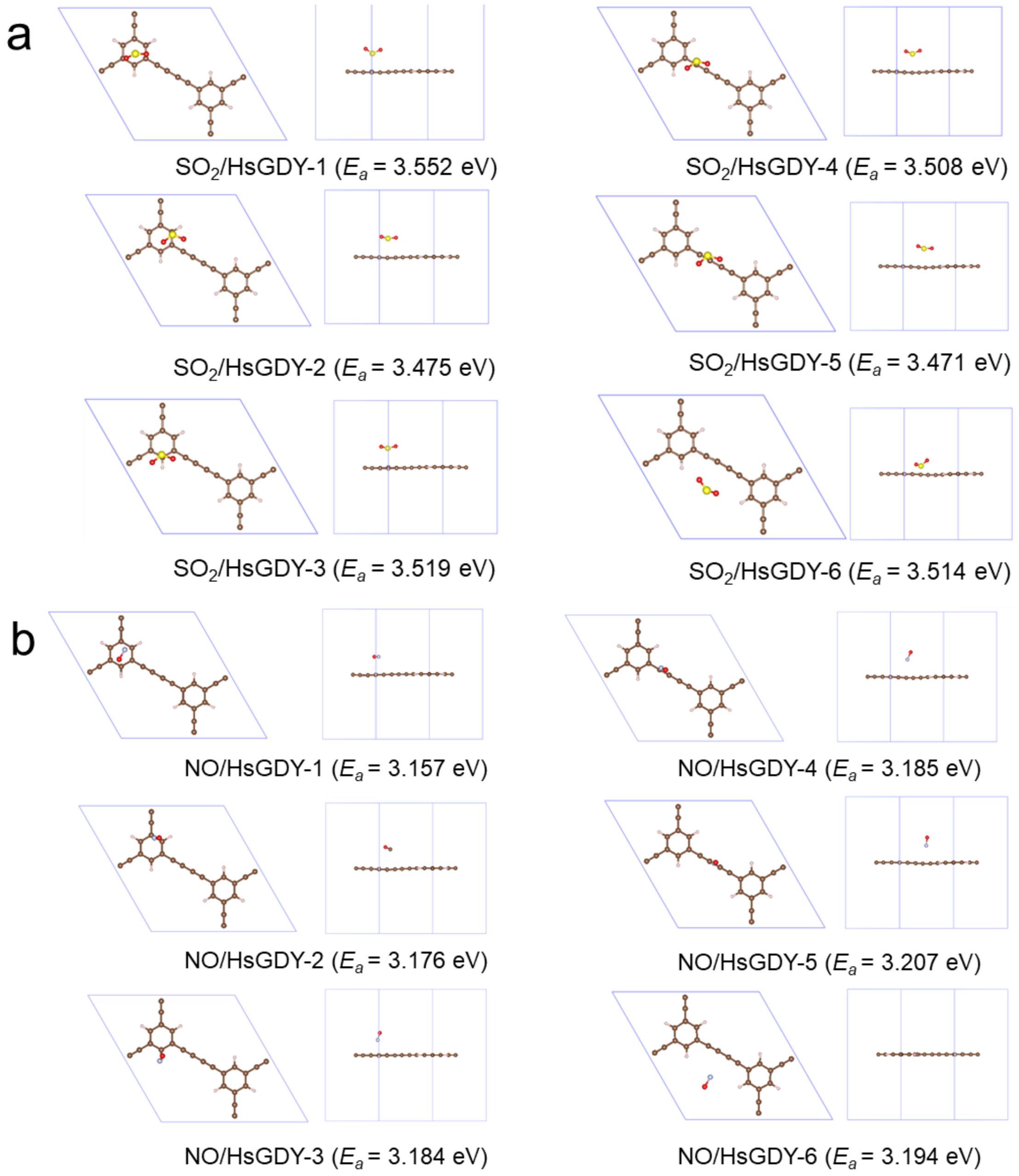

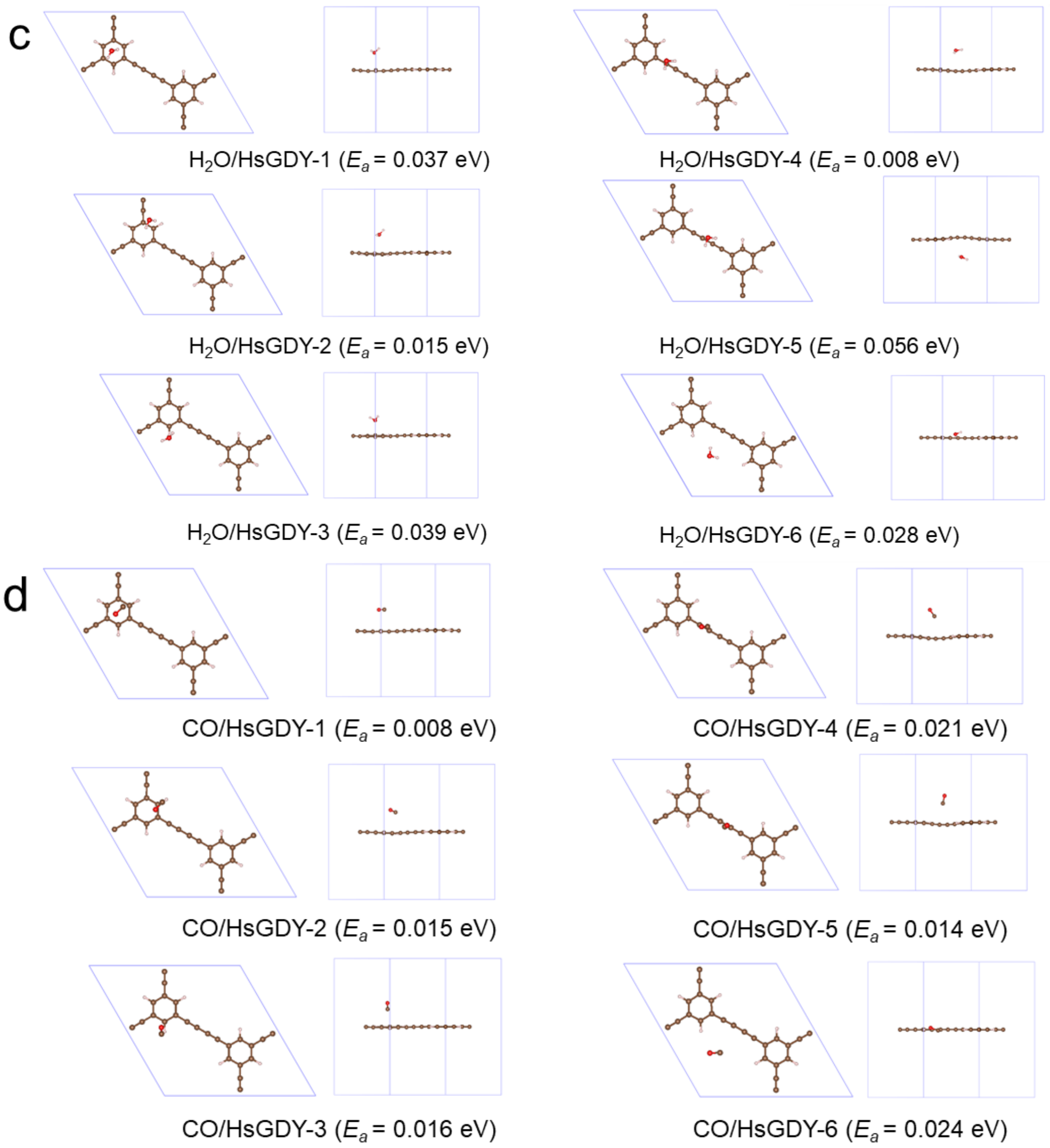
c
H_2O/HsGDY-1 (E_a = 0.037 eV)
H_2O/HsGDY-4 (E_a = 0.008 eV)
H_2O/HsGDY-2 (E_a = 0.015 eV)
H_2O/HsGDY-5 (E_a = 0.056 eV)
H_2O/HsGDY-3 (E_a = 0.039 eV)
H_2O/HsGDY-6 (E_a = 0.028 eV)
d
CO/HsGDY-1 (E_a = 0.008 eV)
CO/HsGDY-4 (E_a = 0.021 eV)
CO/HsGDY-2 (E_a = 0.015 eV)
CO/HsGDY-5 (E_a = 0.014 eV)
CO/HsGDY-3 (E_a = 0.016 eV)
CO/HsGDY-6 (E_a = 0.024 eV)

Supplementary Fig. 12. The schematic diagram of the improved bag filter systems for Hg^0 capture. a HsGDY injection and **b** HsGDY modified filter mediums.

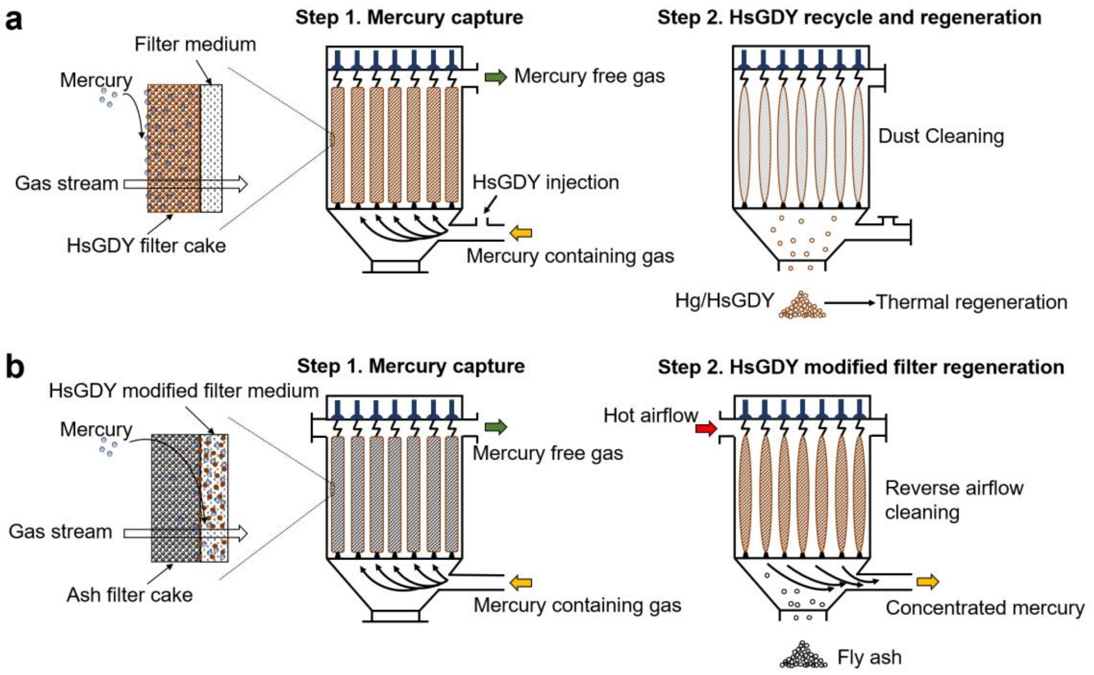

Supplementary Fig. 13. Precipitation experiments. a The image for precipitation particles and **b** the XRD pattern of precipitate.

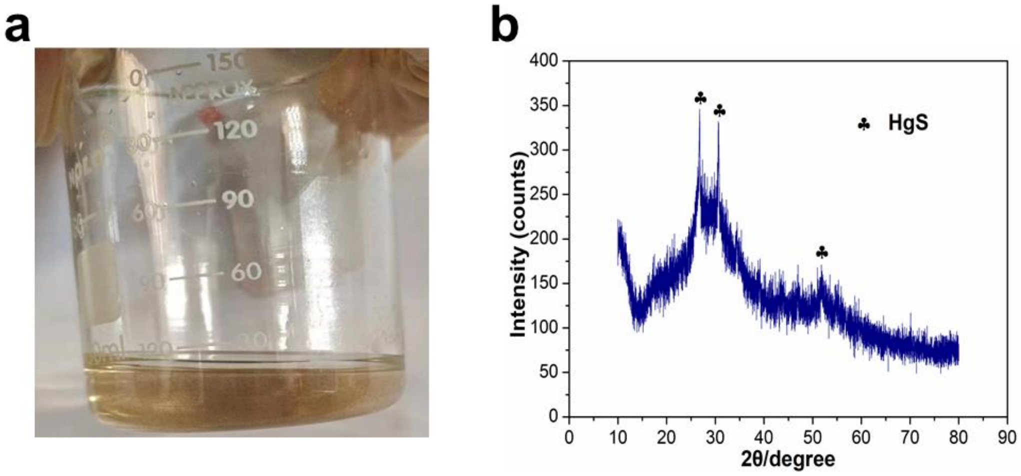

Supplementary Fig. 14. Characterization of HsGDY after Hg^0 adsorption. a The Raman spectra of HsGDY after Hg^0 adsorption (Hg/HsGDY) and **b** the *Hg 4f* XPS analysis of Hg/HsGDY. (The peaks at 1358 and 1579 cm^{-1} are ascribed to aromatic rings. The peaks at 2021 and 2193 cm^{-1} are attributed to acetylenic bonds.)

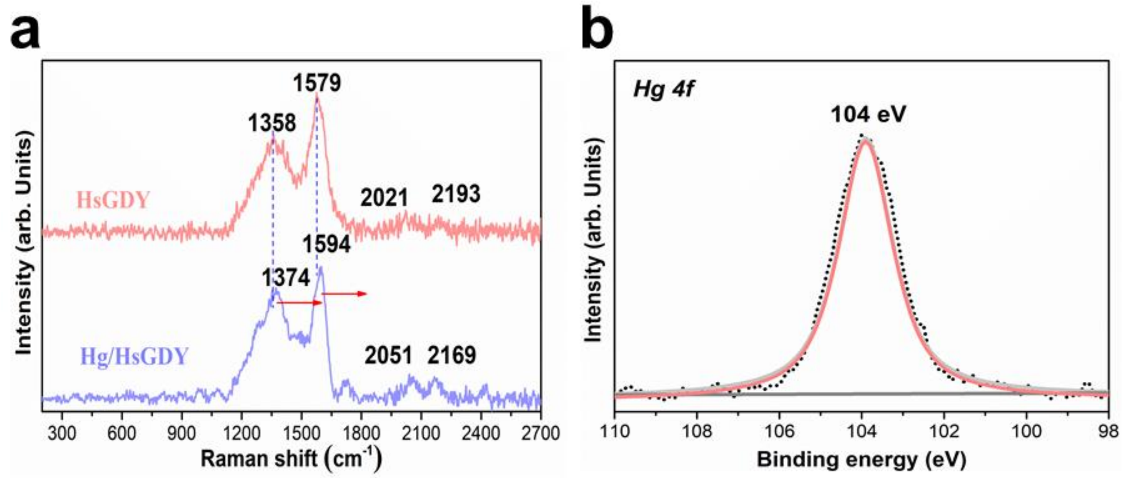

Supplementary Fig. 15. The TEM-mapping results of Hg/HsGDY. a TEM image for Hg/HsGDY, **b** C element distribution, **c** Hg element distribution and **d** energy dispersive X-ray spectroscopy (The insert figures show the enlarged spectra).

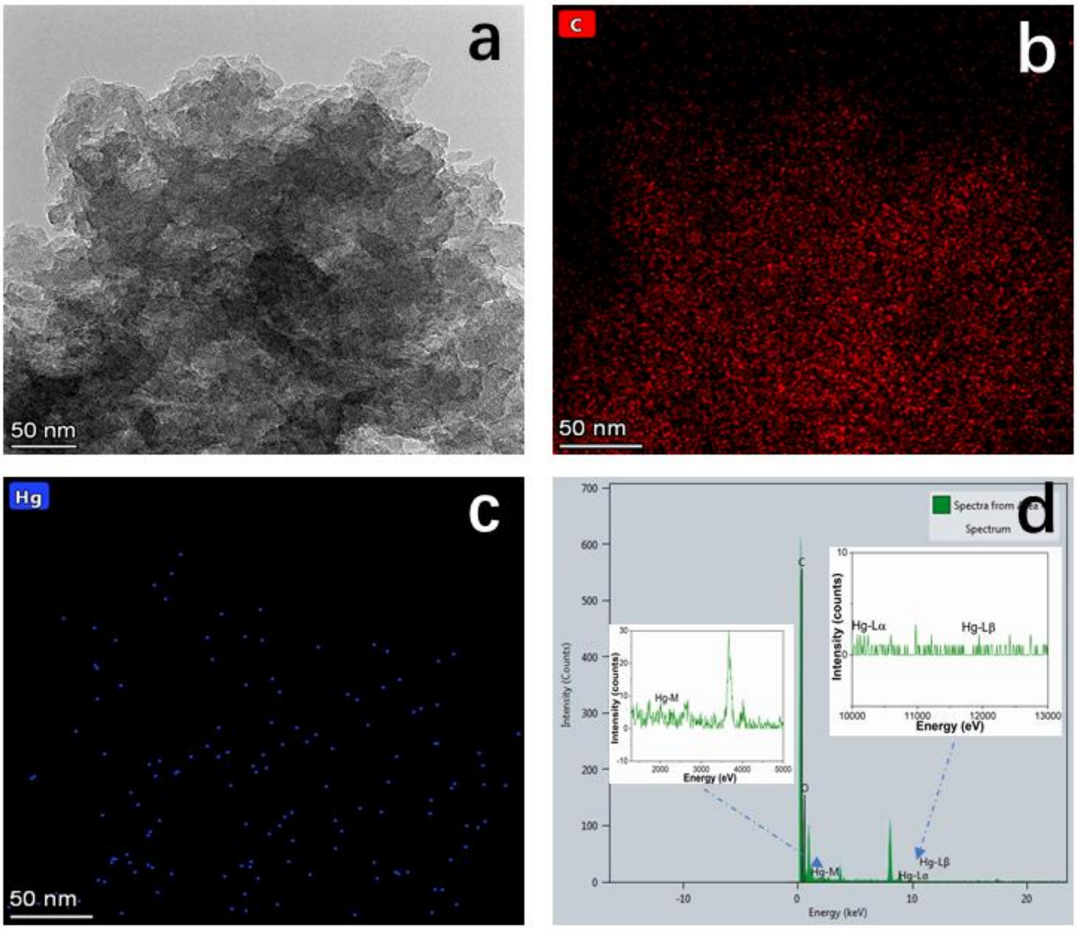

Supplementary Fig. 16. The SEM-EDS results of Hg/HsGDY. a SEM image for Hg/HsGDY and **b** EDS results for Hg/HsGDY (The insert figure shows the enlarged spectrum).

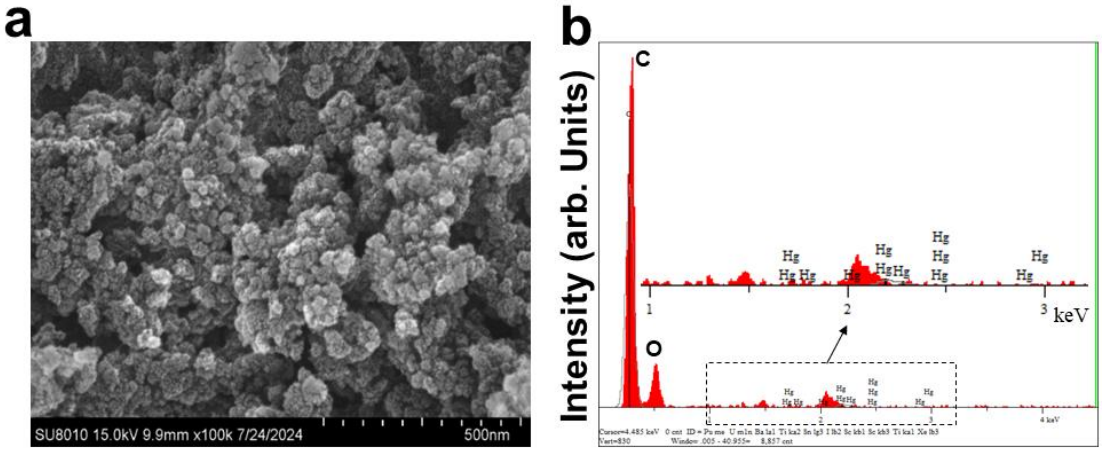

Supplementary Fig. 17. The HAADF-STEM image of fresh HsGDY.

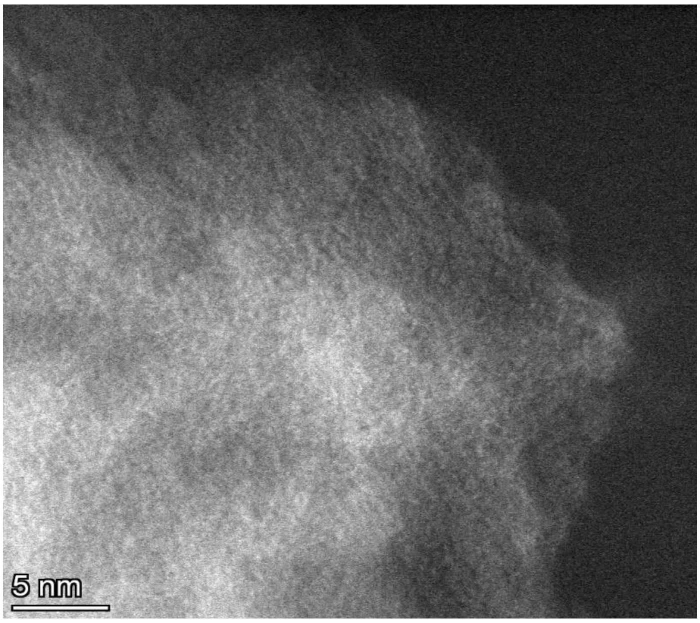

Supplementary Fig. 18. The adsorption process of Hg atom from free state to stable adsorption state over GDY by AIMD simulation (inset figures for the adsorption configurations with time and the resultant electron transfer).

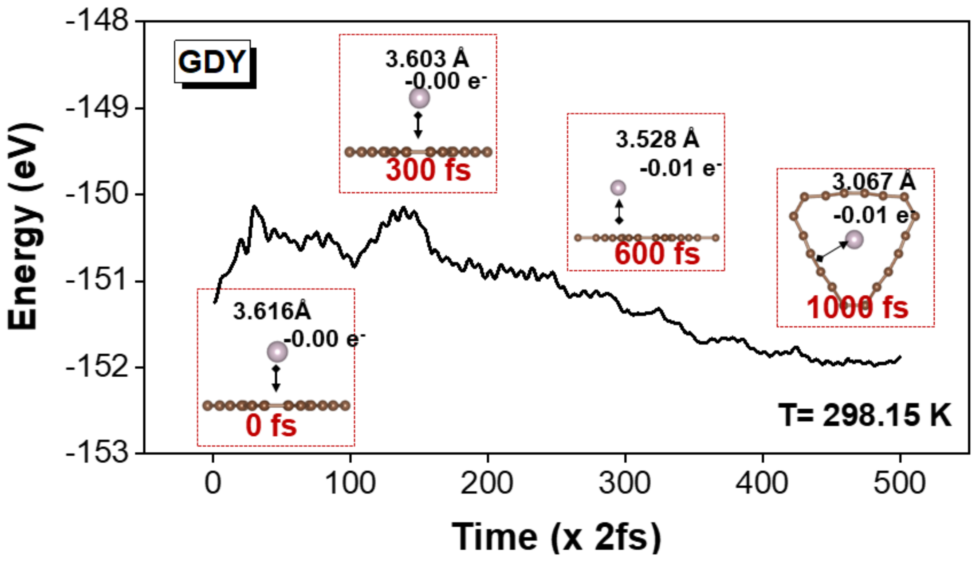

Supplementary Fig. 19. Energy fluctuations after Hg adsorption by AIMD simulation. **a** Hg adsorption onto HsGDY, **b** Hg adsorption onto GDY and **c** Hg adsorption onto GE (inset figures for the adsorption configurations with time and the resultant electron transfer).

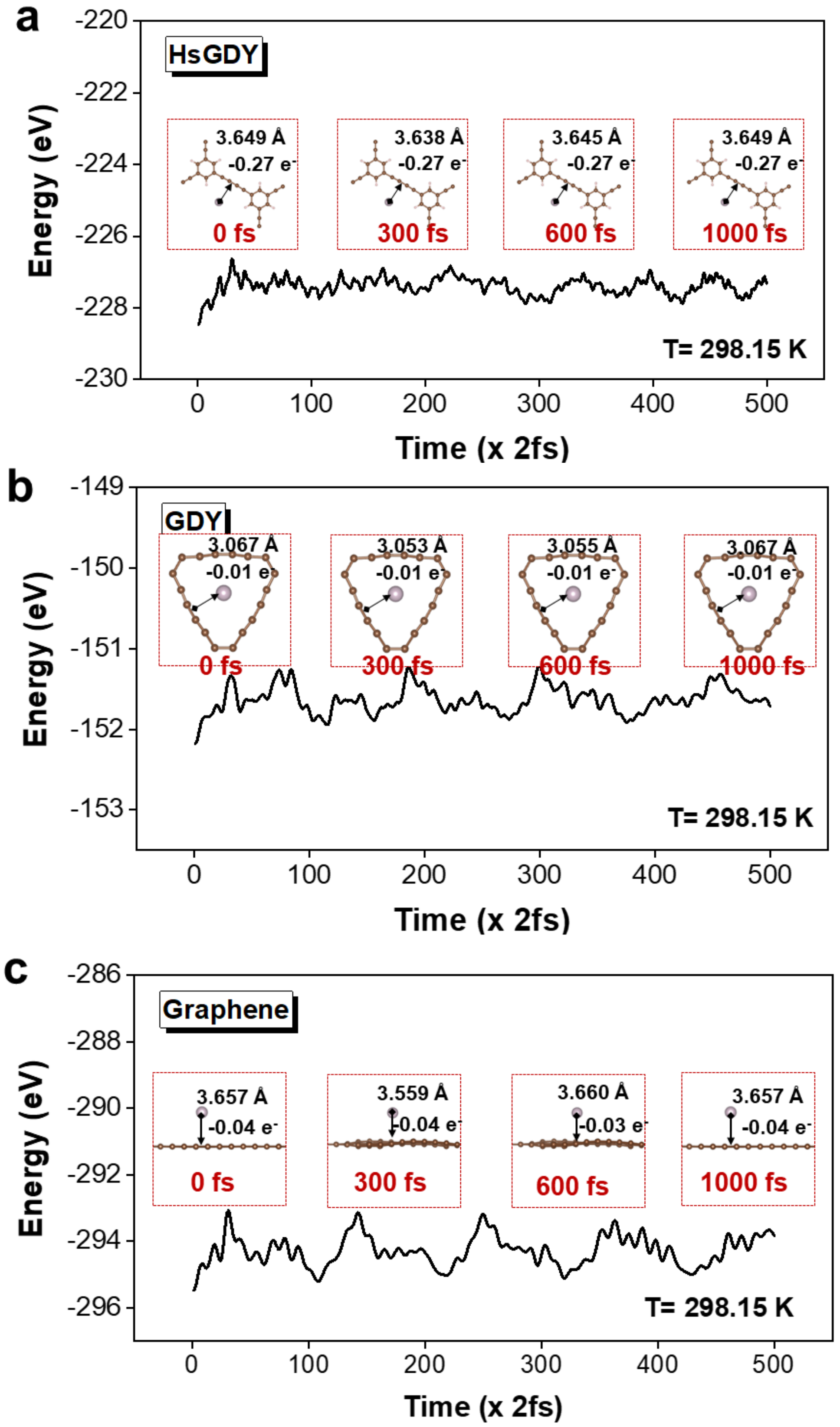

Supplementary Fig. 20. The OHM results of the desorbed mercury species.

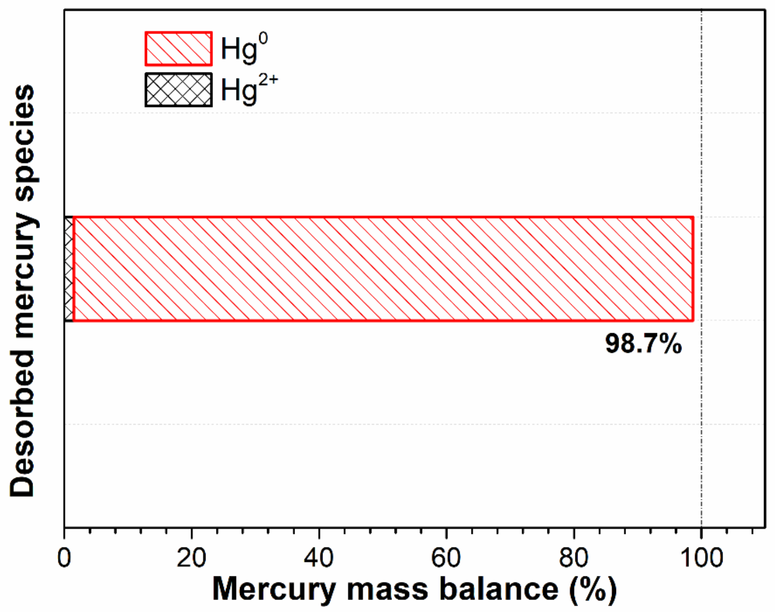

Supplementary Fig. 21. The morphologies and XPS *C 1s* spectra of HsGDY, Hg/HsGDY and HsGDY after regeneration. a *C 1s* spectra and **b** SEM images.

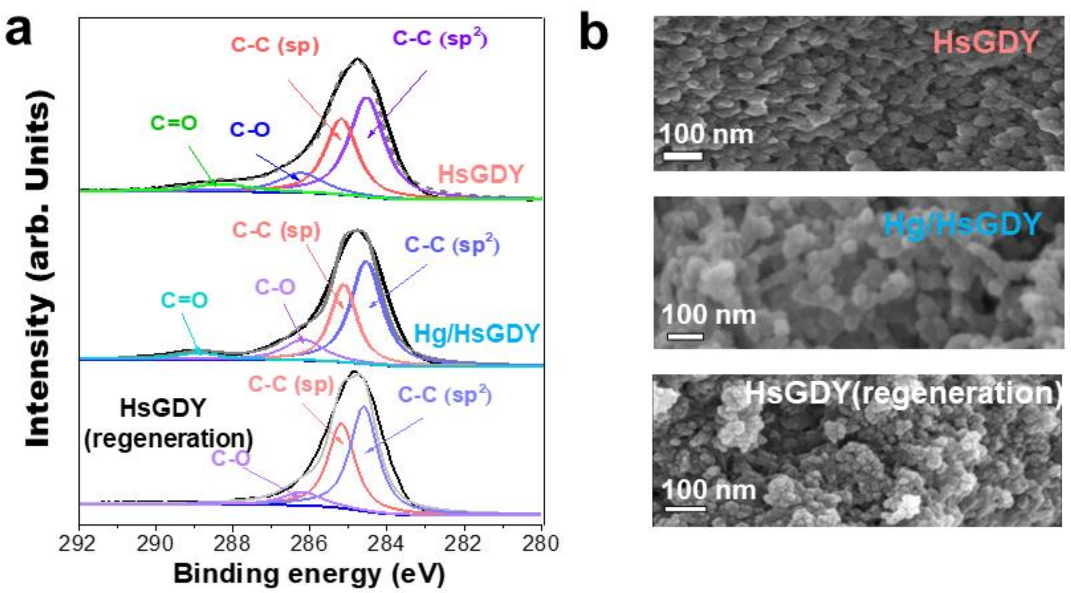

Supplementary Fig. 22. Gibbs free energy of hydrogen evolution for Hg and HsGDY. (The Gibbs free energy of Hg is relatively larger than HsGDY, suggesting that the redox potential of Hg is lower than HsGDY)

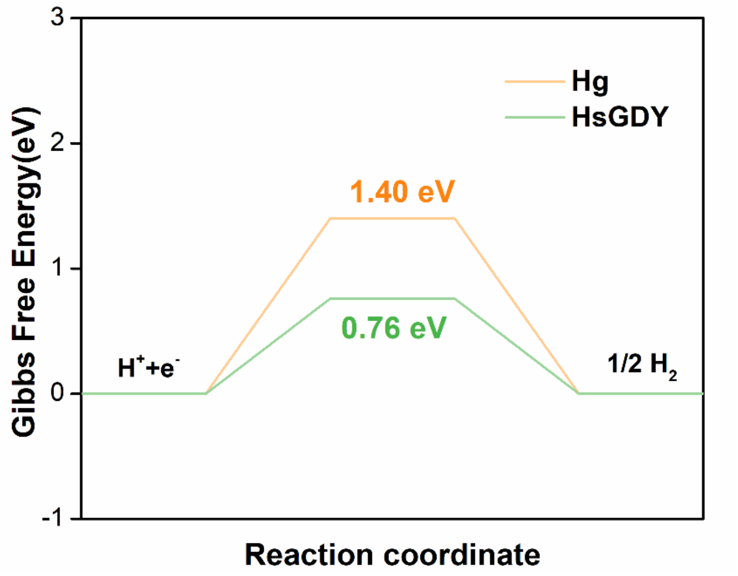

Supplementary Fig. 23. The CV results of the electrochemical experiments.

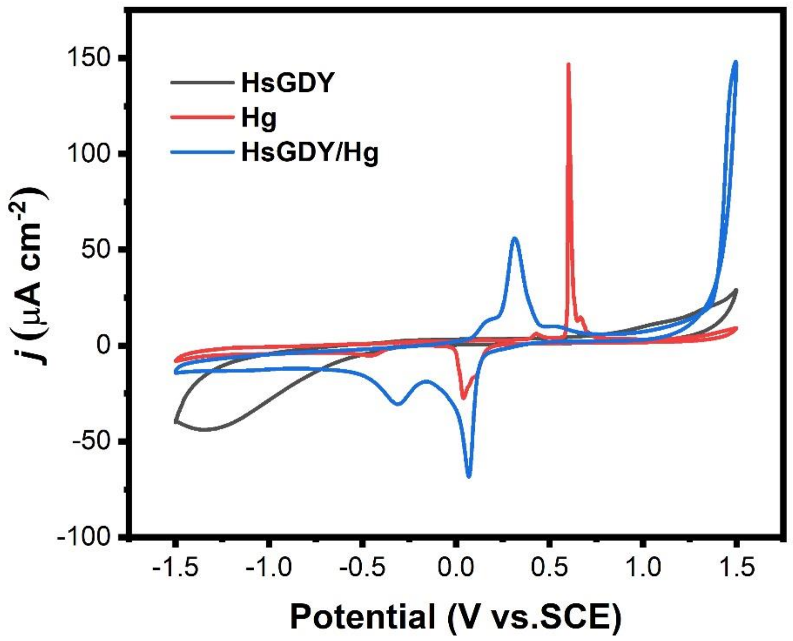

Supplementary Tables

Supplementary Table 1. Experimental conditions.

Experiments	Sample	Gas composition	Temperature
**Set 1.**<br>Performance comparison	GDY/CNT/<br>GE/AC/HsGDY<br>100 mg	340 μg m^{-3} Hg^0<br>N_2	100 °C
**Set 2.**<br>50%-breakthrough curve	HsGDY<br>100 mg	340 μg m^{-3} Hg^0<br>N_2	150 °C
**Set 3.**<br>Effect of adsorption temperature	HsGDY<br>100 mg	340 μg m^{-3} Hg^0<br>N_2	50-200 °C
**Set 4.**<br>Effects of<br>SO_2/NO/H_2O/CO	HsGDY<br>100 mg	340 μg m^{-3} Hg^0<br>0-2400 ppm SO_2<br>0-1000 ppm NO<br>0-3 %vol. H_2O<br>0-800 ppm CO<br>N_2	150 °C
**Set 5.**<br>Performance under simulated flue gas	FMS<br>PTFE<br>HsGDY-FMS<br>HsGDY-PTFE	340 μg m^{-3} Hg^0<br>5 %vol. O_2<br>600 ppm NO<br>600 ppm SO_2<br>3 %vol. H_2O<br>N_2	150 °C

Supplementary Table 2. The possible adsorption configurations and adsorption energies of Hg atom on HsGDY at different sites (in and out of plane sites).

Adsorption Site	Adsorption Configuration (top view)	Adsorption Configuration (side view)	Adsorption Energy (eV)
Hg/HsGDY-1	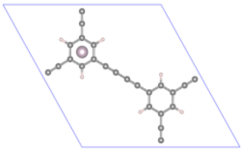	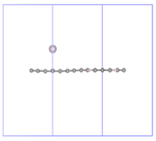	0.012
Hg/HsGDY-2	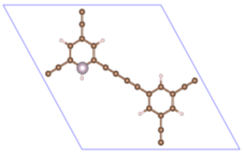	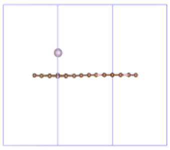	0.041
Hg/HsGDY-3	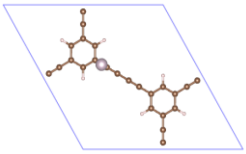	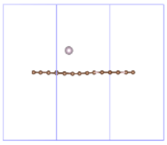	0.032
Hg/HsGDY-4	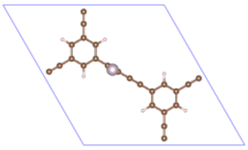	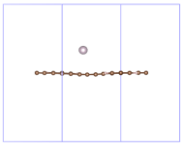	0.015
Hg/HsGDY-5	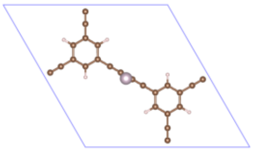	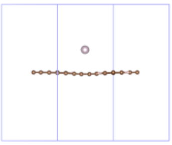	0.043
Hg/HsGDY-6	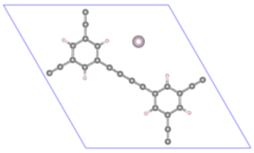	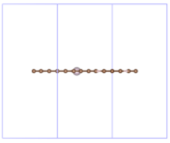	0.012
**Hg/HsGDY-7**	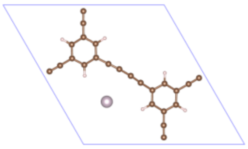	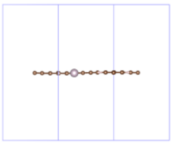	**0.005**

Supplementary Table 3. The possible adsorption configurations and adsorption energies of Hg atom on GDY at different sites (in and out of plane sites).

Adsorption Site	Adsorption Configuration (top view)	Adsorption Configuration (side view)	Adsorption Energy (eV)
Hg/GDY-1	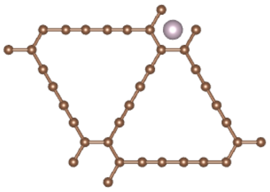	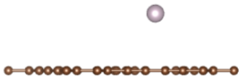	-0.014
Hg/GDY-2	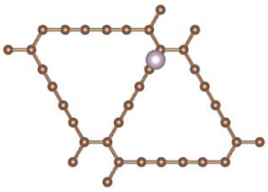	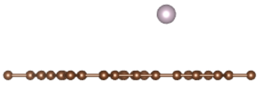	-0.011
Hg/GDY-3	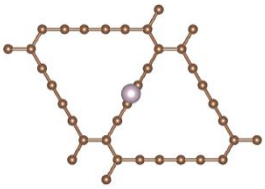	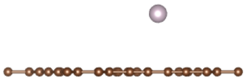	-0.013
**Hg/GDY-4**	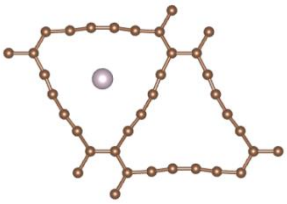	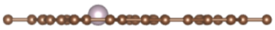	**-0.082**

Supplementary Table 4. The possible adsorption configurations and adsorption energies of Hg atom on GE at different sites (hollow, and top bridge sites).

Adsorption Site	Adsorption Configuration (top view)	Adsorption Configuration (side view)	Adsorption Energy (eV)
Hg/GE-1	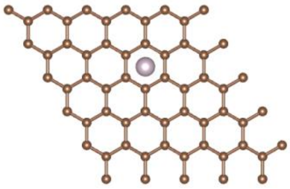	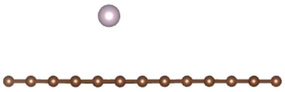	0.124
Hg/GE-2	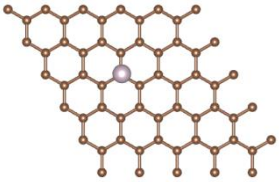	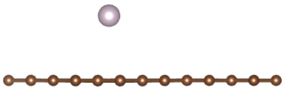	0.125
**Hg/GE-3**	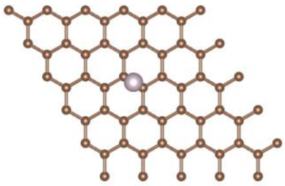	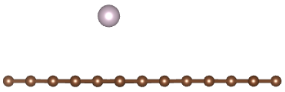	**0.102**

Supplementary Table 5. The possible adsorption configurations and adsorption energies of mercury atom on SWCNT at different sites (inside and outside of hollow, and top bridge sites).

Adsorption Site	Adsorption Configuration (side view)	Adsorption Configuration (top view)	Adsorption Energy (eV)
Hg/SWCNT-1	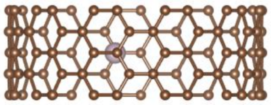	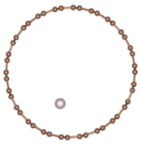	-0.378
Hg/SWCNT-2	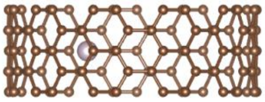	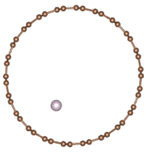	-0.382
**Hg/SWCNT-3**	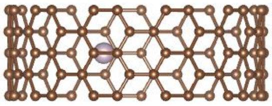	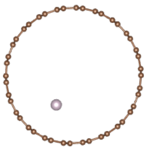	**-0.398**
Hg/SWCNT-4	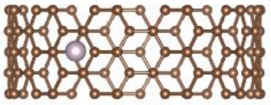	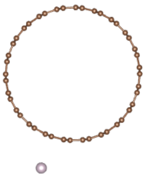	-0.370
Hg/SWCNT-5	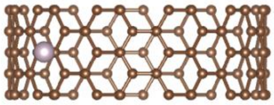	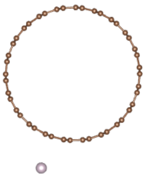	-0.371
Hg/SWCNT-6	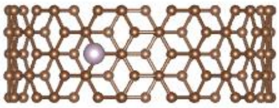	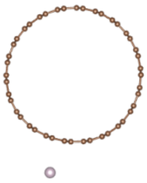	-0.371

Supplementary Table 6. The possible adsorption configurations and adsorption energies of Hg atom on 2HsGDY at different sites.

Adsorption Site	Adsorption Configuration (top view)	Adsorption Configuration (side view)	Adsorption Energy (eV)
**Hg/2HsGDY-1**	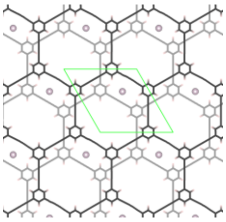	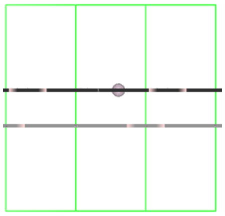	**-0.008**
Hg/2HsGDY-2	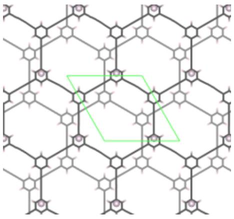	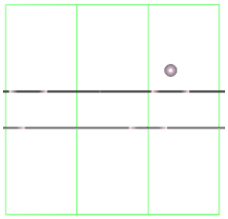	0.007
**Hg/2HsGDY-3**	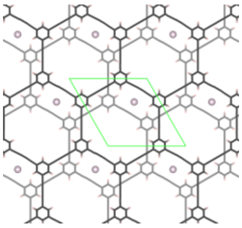	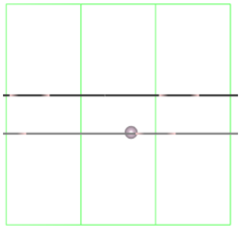	**-0.006**
Hg/2HsGDY-4	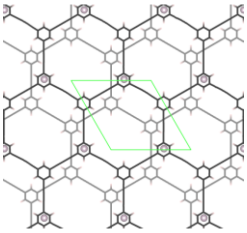	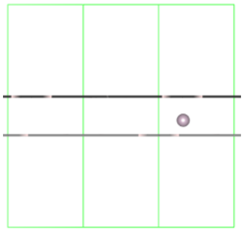	0.031
Hg/2HsGDY-5	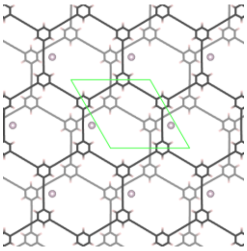	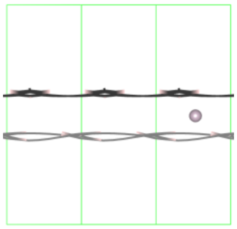	0.079
Hg/2HsGDY-6	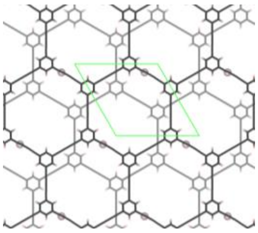	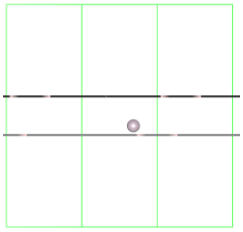	0.011

Supplementary Table 7. The possible adsorption configurations and adsorption energies of Hg atom on 3GDY at different sites.

Adsorption Site	Adsorption Configuration (top view)	Adsorption Configuration (side view)	Adsorption Energy (eV)
**Hg/3GDY-1**	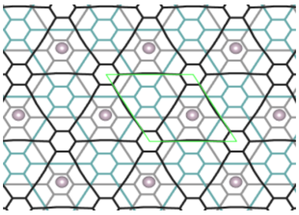	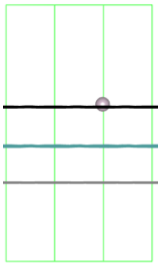	**0.060**
Hg/3GDY-2	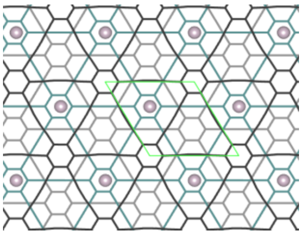	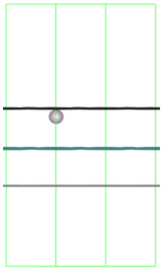	0.095
Hg/3GDY-3	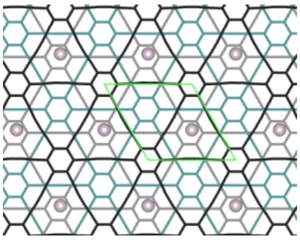	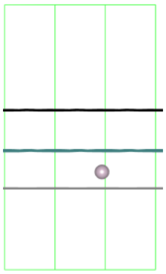	0.081
Hg/3GDY-4	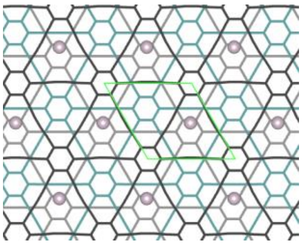	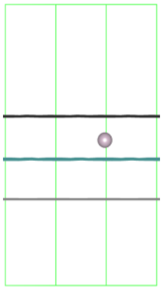	0.087

Supplementary Table 8. The comparison of HsGDY with other carbon sorbents for Hg^0 capture (details can be found in **Supplementary References**).

**Sorbent**	**Hg^0 capture ability**	**Flue gas conditions**	**Sorbent properties**
[1] DARCO® Hg-LH EXTRA	adsorption capacity = 0.471 mg g^{-1} at 423K	80 μg m^{-3} Hg^0, 600 ppm SO_2, 300 ppm NO, 40 ppm HCl, 6% H_2O, 6% O_2 and balance N_2; total flow rate = 1000 ml min^{-1}	commercial AC Br modification
[2] activated carbon A	adsorption capacity = 0.421 mg g^{-1} at 323K	0.35 ng cm^{-3} Hg^0 and balance N_2; total flow rate = 200 cm^3 min^{-1}	carbon prepared from pine wood
[3] AC107-600S	adsorption capacity ≈ 1.6 mg g^{-1} at 150 °C	50 μg m^{-3} Hg^0, 12% CO_2, 1600 ppm SO_2, 50 ppm HCl, 6% O_2 and 7% H_2O; total flow rate = 1000 ml min^{-1}	mixing elemental sulfur with carbon prepared from bituminous coal
[4] AC-Br	adsorption capacity ≈ 2.3 mg g^{-1} at 150 °C	Hg^0 and balance N_2; total flow rate = 2000 ml min^{-1}	AC impregnated with NH_4Br
[5] Ag/graphene	adsorption capacity = 2.4 mg g^{-1} in 100 min at 25 °C	0.5 mg m^{-3} Hg^0 and balance N_2; total flow rate = 500 ml min^{-1}	graphite oxide modified by Ag particles
[6] T6W1P1	adsorption capacity ≈ 0.353 mg g^{-1} at 140 °C	90 μg m^{-3} Hg^0 and balance N_2; GHSV = 209790 h^{-1}	one-step pyrolysis of plastic and wood
[7] RS-S	adsorption capacity ≈ 0.127 mg g^{-1} at 30 °C	20 μg m^{-3} Hg^0 and balance N_2; total flow rate = 1000 ml min^{-1}	rice straw pyrolysis followed by H_2S plasma modification
[8] T5CUF0.3	adsorption capacity ≈ 0.106 mg g^{-1} in 60 min at 200 °C	90 μg m^{-3} Hg^0, 20% H_2, 20% CO, 10 ppm HCl, 400 ppm H_2S and balance N_2; GHSV = 50000 h^{-1}	tea biomass carbon modified by FeO_X
[9] RPC-313	adsorption capacity ≈ 0.797 mg g^{-1} at 120 °C	33 μgm^{-3} Hg^0, 50 ml min^{-1} O_2 and 1450 ml min^{-1} N_2;	prepared from rice straw and PVC powder

		total flow rate = 1500 ml min^{-1}	
[10] BC900	adsorption capacity ≈ 0.036 mg g^{-1} at 100 °C	90 μg m^{-3} Hg^0, 6% O_2, 300 ppm NO, 600 ppm SO_2, 40 ppm HCl, 10% H_2O and balance N_2; total flow rate = 1000 ml min^{-1}	raw biochar pyrolyzed from wood powder
[11] E8B5	adsorption capacity ≈ 0.304 mg g^{-1} at 130 °C	50 μg m^{-3} Hg^0, 5% O_2, 600 ppm SO_2, 400 ppm NO, 1.5% H_2O and balance N_2; GHSV = 10000 h^{-1}	NH_4Br modified biochar
[12] ACF	adsorption capacity ≈ 0.65 mg g^{-1} at 25 °C	21 ng mL^{-1} Hg^0 and air	activated carbon fibers
HsGDY (this work)	adsorption capacity ≈ 1.27 mg g^{-1} at 150 °C	340 μg m^{-3} Hg^0 and balance N_2; GHSV = 53000 h^{-1}	pure carbon material excellent regeneration property excellent SO_2 resistance

Supplementary Notes

The laws of variation of Hg^0 adsorption capacity with average pore size and pore volume are exhibited in **Supplementary Fig. 6**. It can be seen that there is some correlation between the Hg^0 adsorption performance and the pore characteristics. But the pore volume or pore size is not perfectly consistent with the Hg^0 adsorption performance. An inverted U curve can be observed. For an example, the pore volume of HsGDY is 0.3758 cm^3/g, which is obviously lower than that of CNT and AC. However, the Hg^0 adsorption performance of HsGDY is evidently better than CNT and AC. This is because adsorption is a complex process that involves many factors beyond just the pore volume and pore size. As mentioned in the text, the capture of Hg^0 onto HsGDY is benefited from the large hexagonal pore structure and surface charge heterogeneity of HsGDY. These unique properties of HsGDY can lead to a strong interaction of HsGDY with Hg atoms, even if the BET surface area as well as pore volume of HsGDY is not the highest.

The oxidation of Hg over HsGDY was also investigated by electrochemical experiments. The results are displayed in **Supplementary Fig. 23**. The peaks at 0.42 eV and 0.61 V for the Hg electrode is attributed to the oxidation peaks of $Hg^0 \rightarrow Hg^+$ and $Hg^+ \rightarrow Hg^{2+}$, respectively. The existence of HsGDY leads to the peaks shifting to the lower voltage direction. On the HsGDY/Hg electrode, the Hg atoms have been existed in a partially oxidized state. The Hg atoms on HsGDY enjoy a shorter path to be oxidized into Hg^{2+}. The oxidation peak area for the HsGDY/Hg electrode is much larger than Hg electrode. This result indicates that HsGDY promotes the oxidation of Hg. The reduction peak area for the HsGDY/Hg electrode is obviously larger than its oxidation peak. The reason is that the amount of electron transfer for $Hg^{2+} \rightarrow Hg^0$ is larger than Hg (partially oxidized state) $\rightarrow Hg^{2+}$.

Supplementary References

[1] Yang, X. et al. Adsorption and catalytic oxidation of elemental mercury over regenerable magnetic Fe-Ce mixed oxides modified by non-thermal plasma treatment. *Chem. Eng. J.* **358**, 1454-1463 (2019).

[2] Skodras, G., Diamantopoulou, I., Zabaniotou, A., Stavropoulos, G. & Sakellaropoulos, G. P. Enhanced mercury adsorption in activated carbons from biomass materials and waste tires. *Fuel Process. Technol.* **88** 749-758 (2007).

[3] His, H. C., Rood, M. J., Rostam-Abadi, M. & Chang, Y. Effects of sulfur, nitric acid, and thermal treatments on the properties and mercury adsorption of activated carbons from bituminous coals. *Aerosol. Air Qual. Res.* **13**, 730-738 (2013).

[4] Zhou, Q. et al. Experimental and kinetic studies of gas-phase mercury adsorption by raw and bromine modified activated carbon. *Fuel Process. Technol.* **134**, 325-332 (2015).

[5] Xu, H. et al. Regenerable Ag/graphene sorbent for elemental mercury capture at ambient temperature. *Colloids Surf. A* **476**, 83-89 (2015).

[6] Xu, Y. et al. Development of waste-derived sorbents from biomass and brominated flame retarded plastic for elemental mercury removal from coal-fired flue gas. *Chem. Eng. J.* **350** 911-919 (2018).

[7] Zhang, H. et al. Enhanced mercury removal by transplanting sulfur-containing functional groups to biochar through plasma. *Fuel* **253**, 703-712 (2019).

[8] Altaf, A. R., Adewuyi, Y. G., Teng, H., Gang, L. & Abid, F. Elemental mercury (Hg^0) removal from coal syngas using magnetic tea-biochar: Experimental and theoretical insights. *J. Environ. Sci.* **122**, 150-161 (2022).

[9] Shi, Q. et al. Enhanced elemental mercury removal via chlorine-based hierarchically porous biochar with $CaCO_3$ as template. *Chem. Eng. J.* **406**, 126828 (2021).

[10] Zhou, M. et al. Facile synthesis of phosphorus-doped porous biochars for efficient removal of elemental mercury from coal combustion flue gas. *Chem. Eng. J.* **432**, 134440 (2022).

[11] Xu, W., Pan, J., Fan, B. & Liu, Y. Removal of gaseous elemental mercury using seaweed chars impregnated by NH_4Cl and NH_4Br. *J. Cleaner Prod.* **216**, 277-287 (2019).

[12] Yao, Y., Velpari, V. & Economy, J. Design of sulfur treated activated carbon fibers for gas phase elemental mercury removal. *Fuel* **116**, 560-565 (2014).
