# Supplementary material for: In-situ adsorption-coupled-oxidation enabled mercury vapor capture over sp-hybridized graphdiyne
Source: Nat Commun. 2025 Mar 11;16:2439. doi: 10.1038/s41467-025-57197-8 (PMC11897331; doi:10.1038/s41467-025-57197-8)
Supplement: Supplementary file 2 — Description of Additional Supplementary Files [file 41467_2025_57197_MOESM2_ESM.pdf]

Description of Additional Supplementary Files

File name: Supplementary Data 1-20
Description:

1 AIMD source data (Final state) for Supplementary Fig. 18 (The adsorption process of Hg atom from free state to stable adsorption state over GDY by AIMD simulation)

2 AIMD source data (Final state) for Supplementary Fig. 19b (Energy fluctuations after Hg adsorption onto GDY by AIMD simulation)

3 AIMD source data (Initial state) for Supplementary Fig. 18 (The adsorption process of Hg atom from free state to stable adsorption state over GDY by AIMD simulation)

4 AIMD source data (Initial state) for Supplementary Fig. 19b (Energy fluctuations after Hg adsorption onto GDY by AIMD simulation)

5 AIMD source data (Final state) for Supplementary Fig. 19c (Energy fluctuations after Hg adsorption onto GE by AIMD simulation)

6 AIMD source data (Final state) for Fig. 4k (The molecular dynamic simulation results of Hg adsorption over GE

7 AIMD source data (Initial state) for Supplementary Fig. 19c (Energy fluctuations after Hg adsorption onto GE by AIMD simulation)

8 AIMD source data (Initial state) for Fig. 4k (The molecular dynamic simulation results of Hg adsorption over GE

9 AIMD source data (Final state) for Fig. 4j (The molecular dynamic simulation results of Hg adsorption over HsGDY

10 AIMD source data (Final state) for Supplementary Fig. 19a (Energy fluctuations after Hg adsorption onto HsGDY by AIMD simulation)

11 AIMD source data (Final state) for Fig. 5d (Hg desorption from HsGDY)

12 AIMD source data (Initial state) for Fig. 4j (The molecular dynamic simulation results of Hg adsorption over HsGDY

13 AIMD source data (Initial state) for Supplementary Fig. 19a (Energy fluctuations after Hg adsorption onto HsGDY by AIMD simulation)

14 AIMD source data (Initial state) for Fig. 5d (Hg desorption from HsGDY)

15 Source data for Fig. 1b (2D charge distribution of HsGDY)

16 Source data for Fig. 4e (The two-dimensional projection of differential charge density contours of Hg/HsGDY)

17 Source data for Fig. 4d (The charge density difference of Hg/HsGDY)

18 Source data for Supplementary Fig. 1b (2D charge distribution of GDY)

19 Source data for Fig. 4g (The charge density difference of Hg/GE)

20 Source data for Fig. 4h (The two-dimensional projection of differential charge density contours of Hg/GE)
